# Anesthesia and Analgesia in Laboratory Animals, Third Edition

**DOI:** 10.1017/awf.2023.90

**Published:** 2023-10-23

**Authors:** Lucy Whitfield

**Affiliations:** OWL Vets Ltd, Mildenhall, UK

This is the third edition of this comprehensive review of anaesthesia and analgesia sponsored by the American College of Laboratory Animal Medicine and is intended for all those involved in biomedical research, particularly veterinarians, those conducting the research and animal welfare committees.

The editors are to be congratulated for bringing together over 70 globally eminent individuals to produce the 30 chapters of this informative manual, giving a wide perspective on animals, agents and anaesthetic use within research.

New in this edition is consideration of some ethical aspects of anaesthetic use, which set the context, whilst acknowledging that it starts from the premise that use of animals in research and teaching is permissible. Legislative approach and regulatory frameworks in different parts of the world are compared constructively, together with suggestions for developing a local (institutional) policy framework, which will be of particular help and interest for ethical and scientific review committees.

The next section provides an excellent overview of sedation, anaesthesia and analgesia, including pharmacology of both injectable and inhaled agents and detail of their action on receptors. A huge variety of drugs is listed, including some of historical interest or non-pharmaceutical quality, with comprehensive information for the reader on effects, adverse effects and routes of administration. The authors explain the importance of using sedatives as part of a balanced anaesthesia protocol and provide information to assist the rational choice of agent.

Interestingly, subcutaneous injection is not included as a route of administration of injectable anaesthetics, although successfully used in both clinical and research settings. This chapter would also be a good opportunity to remind the reader about the importance of providing oxygen to prevent hypoxia during anaesthesia using injectable agents. Examples of drug pharmacokinetics, metabolism and excretion are given, however it’s not always clear to which species they refer, and practical doses are not always provided which can be a little confusing for the reader who is trying to select the most appropriate agent, route and dose. However, doses are provided later in the book, so this may simply be a result of preventing repetition.

The chapter on inhaled anaesthetics includes an extensive section on operator safety, which is very much oriented to USA regulations, although the principles of safe anaesthetic use apply more widely. This thorough chapter includes examples of anaesthetic workstations and multi-animal anaesthetic delivery systems. The need for regular testing and servicing for anaesthetic equipment is highlighted.

Neuromuscular blocking agents (NMBA) are considered, together with caveats, including robust scientific justification for their use. The anaesthetic regime must be reliable, with adequate physiological monitoring, ventilation and monitoring of blockade. The authors note the potential use of ultra-short-acting and/or reversible drugs which will increase the versatility and safety of NMBA use.

Section three sets out practical information on delivery and monitoring for anaesthesia, including a clear explanation and illustrations of circuits and delivery systems available. Open and closed systems are explained, including use of face masks, endotracheal tubes and supraglottic devices at the patient end. There is also a useful section on the different types and uses of mechanical ventilators. A notable omission, though, is discussion about low-flow inhalation systems for small rodents, which have advantages including the reduction of waste gas and less chilling of the patient.

Anaesthesia disrupts the animal’s homeostasis, and this may be exacerbated by the long procedures applied in research, or when anaesthetising animals with pre-existing pathology. Chapter 10 emphasises that the first principle of monitoring anaesthesia is to assure the welfare of the animal and secondly to interpret correctly the physiological changes that may occur, before intervening appropriately. Core components of monitoring are set out; from basic physical observations to use of physiological monitoring machines. The authors consider too the challenges and considerations for effective monitoring in small laboratory animals.

Continuing the consideration of care of research animals, the chapter on peri-procedural care starts with emphasis on the importance of proper oversight, good planning, consultation with veterinary experts and consideration of the needs of the species involved. Helpful guidance, such as PREPARE Guidelines are cited as a useful checklist, as well as a sample ‘animal surgery’ checklist for daily use. The chapter is written from a helpful and practical standpoint, including consideration of the importance of meeting suitable housing and psychological needs of small rodents, larger species and agricultural animals. This chapter is nicely illustrated, with examples of monitoring, personnel training, specific patient care, nutritional support and humane endpoints, should the procedure or recovery not go as planned.

The legal and societal reasons for pain prevention in these sentient animals are discussed in chapter 12, together with the challenges of effective pain assessment in the laboratory, especially as regards the prey species that make up the majority of animals used. Effective assessment of pain is crucial to pain management and this chapter sets out logical and clear indications for timing, methods and documentation of assessment. Validated pain measures, as well as the challenges of pain assessment, are set out and with a suggested solution that home-cage automated assessment tools, such as activity and/or video monitoring, are useful tools both to reduce the subjectivity of observers and to address the need to monitor these nocturnal animals during their active phase.

Section 5 contains 13 separate chapters and includes a wide range of species and classes of animal, ranging from mammals, avians and amphibia to marsupials, fish and invertebrates. The commonly used lab animal species are covered in detail, including consideration of species-specific anatomy in relation to anaesthesia and practical considerations for anaesthesia and analgesia.

An extensive chapter on laboratory rodents fits with their place as the most commonly used animals in research. The authors are to be commended on their pragmatic approach to setting out current best practice in rodent anaesthesia with discussion of methods that are reliable, safe and relatively straightforward for the non-expert anaesthetist to perform. The need for careful planning at the pre-procedural stage is emphasised, including deciding on the required degree and nature of anaesthesia, patient evaluation, and for provision of analgesia. A number of anaesthetic agents are discussed, together with helpful tables of dose rates, route and references. The chapter includes anaesthesia for specific situations and managing common anaesthetic emergencies, as well as a thorough section on post-operative care and rational use of analgesics. Again, a helpful table of agents, doses and references is included. The section on approaches to analgesia mentions several methods of pain assessment and suggests that a combination of methods is most likely to produce accurate results. Given the utility and uptake of facial grimace scales in a variety of species, it seems odd that this is not signposted here, as it may be a challenge for the less experienced reader to select appropriate method(s) and interpret signs of pain. Rodents other than rats and mice are considered separately and the relevant species differences are made clear.

The chapter on rabbits has been updated from the previous edition and provides an excellent overview of anaesthesia and analgesia in the species. The authors also direct the reader to other journals for up-to-date information as practices change. Available anaesthetic and analgesic agents are listed, together with suggested delivery systems, including endotracheal tubes and supra-glottic devices. Annotated line drawings and photos add clarity to the text. Options for anaesthetic monitoring and patient support are discussed and illustrated, together with information about optimising patient care.

Fish encompass a huge variety of species, although there are fewer that are routinely used in the research setting. Again, pre-procedural planning is emphasised encompassing human as well as fish safety. Physical restraint as well as anaesthesia is discussed, including the use of neuromuscular blockers or cooling to immobilise fish, as well as electro-fishing outside the laboratory, where the reader is referred to their local ethical review committee before proceeding. Anaesthetic and analgesic agents are considered, with examples for different species. The challenges of post-operative care, including avoiding stress, assessment of pain and control of any adverse effects. A table of welfare assessment parameters is included to guide the reader.

The chapter on pigs is particularly thoughtful, written from both a practical and welfare-oriented standpoint, pointing out the need to understand the pig’s natural behaviour and to work with the animal through training in order to reduce stress. Precise and practical instruction on basic physiology involved in anaesthesia is provided which seeks to improve stability during the procedure, as well as manage the procedure for better patient (and scientific) outcomes. The authors emphasise the need for researchers to take expert advice prior to undertaking anaesthesia, particularly if neuro-muscular blockade is being considered. This chapter also includes some excellent photographic illustrations of the points in the text.

Anaesthesia and analgesia of non-human primates (NHPs) is covered comprehensively, although very much from a US standpoint, with availability of drugs in this country, such as dexmedetomidine, rather than the racemic mixture used elsewhere in the world. The chapter includes helpful tables of doses for anaesthetics and analgesics, although in some cases the routes of administration for the analgesics are regrettably omitted. Some more information on monitoring and maintenance of homeostasis during anaesthesia would perhaps be helpful, particularly as the larger NHPs are often used in long-lasting anaesthetic and surgical procedures. There is a well-illustrated section on placement of nerve blocks in the head, as well as sections on paediatric and geriatric anaesthesia in NHPs.

Various breeds of dog and cat are considered in chapter 19, whereas most research texts would consider only the beagle dog. There is particular emphasis on good preparation of equipment, drugs and personnel before anaesthesia, including a pre-anaesthetic checklist. This should be applied to all anaesthesia situations and is worth repeating and emphasising elsewhere too (e.g. in the principles of anaesthesia section).

It’s great to see a separate chapter on ferret anaesthesia and analgesia, as specific information can be challenging to find in other laboratory texts – the species’ differences and tips for care are pertinent and practical.

The chapter on ruminant anaesthesia includes a helpful discussion on fasting prior to anaesthesia, as there are different opinions and practices in place; the effects of fasting are explained, helping the reader to make a more informed decision. It is emphasised that ruminants are prey animals and thereby need to be handled accordingly during pre- and post-operative care, for example not undergoing isolation from herd-mates and having their analgesic needs properly met. Important safety considerations, such as ensuring that ruminants are intubated and never anaesthetised in dorsal recumbency are highlighted.

A welcome addition to this book is a chapter collecting together information on anaesthesia concerning a variety other mammals that may be used in research, including chinchillas, naked mole rats, bats and armadillos. This really does make the ACLAM manual a ‘one-stop-shop’ for everything anaesthesia.

Species-specific anatomy and physiology are reprised in the avian chapter, including explanation of the role of the air sacs in breathing and advice on positioning of the bird during anaesthesia. Large and small species are considered, including sedation for more challenging species, such as ratites.

There’s a huge diversity of over 10,000 species of reptiles, representing a formidable challenge for a single chapter! Important issues, such as the circulatory shunts in many species are emphasised, which must be taken into account if injectable anaesthetic and analgesic agents are used. The chapter includes tables of dose rates and routes for sedative, anaesthetic and analgesic agents for various species, as well as advice on post-operative care and pain assessment.

Amphibia are another large class of species but both terrestrial and aquatic species are well considered in chapter 25. Specific anatomical features, such as their delicate skin, are emphasised, along with consideration of the available anaesthetic agents and recommendations for specialised procedures.

The final species chapter considers invertebrates – again a huge range, including insects, arachnids, molluscs and decapods. The differing legal protection of cephalopods and decapods, which are important research subjects, in the USA and, for example, EU is highlighted; the authors recommend that, whatever the regulatory situation, a humane approach to use and consideration of stress responses should always be applied.

The final section considers a variety of special topics for anaesthetic and analgesic use, tackling poorly understood issues such as chronic (maladaptive) pain. The sequelae of long-standing pain are set out, together with our current poor ability to detect and evaluate maladaptive pain in most non-human species and some treatments are listed.

Anaesthesia for pregnant animals, foetuses and neonates is examined in chapter 28, with recommendations for supportive care of neonatal animals, post anaesthesia (and surgery). Interestingly, hypothermia is still included as a permitted method of ‘restraint’ of neonatal animals, although the data regarding the adverse effects of this procedure are also presented.

The imaging of animals during experiments is a commonly used procedure; the chapter in this book provides an excellent summary of the modalities in use. The authors explain clearly how the quality and stability of the animal’s physiological state (particularly common anaesthetic problems such as hypoxia and hypothermia) impact significantly on the quality of the data gained and the reproducibility of the study. The authors stress the need for researchers to seek expert advice regarding the choice and application of the anaesthesia and understand the effects that different agents will have on the animal, and therefore the data.

The final chapter of the book reviews the use of animals in pain research, with discussion of the available Non-Animal Methods (NAMs) that may provide a more robust and mechanistic approach to some scientific questions, as well as the current animal models of pain that are in use. The authors suggest that use of technology will assist researchers in better understanding the animals’ response to pain (and pain models); much of the pain research is carried out in nocturnal small rodents, so use of automated monitoring systems, camera recording and computational analysis will produce a much more robust and reliable dataset than periodic elicited observations.

Overall, this is an excellent, well-written and comprehensive book of 760 pages, with a great deal of information and detail in a form that is accessible to both veterinarians and researchers. The list of contents contains details of sections and chapters to signpost the reader directly and is complemented by a short index at the back of the book.

That said, readers should take the time first to review the principles of anaesthesia and analgesia, as well as the species-specific sections, as the two are intended to be complementary. The book includes consideration of both the use of anaesthesia and analgesia for humane purposes but also information to help the researcher select the most appropriate agent with regard to the collection of reproducible and reliable scientific data. It’s an up-to-date and essential addition to the reference library for any institution that uses animals in research.

